# Genome Sequence of the AIDS-Associated Pathogen Penicillium marneffei (ATCC18224) and Its Near Taxonomic Relative Talaromyces stipitatus (ATCC10500)

**DOI:** 10.1128/genomeA.01559-14

**Published:** 2015-02-12

**Authors:** William C. Nierman, Natalie D. Fedorova-Abrams, Alex Andrianopoulos

**Affiliations:** a The J. Craig Venter Institute, Rockville, Maryland, USA; b Department of Genetics, University of Melbourne, Victoria, Australia

## Abstract

Penicillium marneffei can cause a fatal systemic mycosis in patients infected with the HIV. Infections are endemic in the tropical regions of southeast Asia. Here, we report the genome sequences of the type strains of P. marneffei and its avirulent near relative, Talaromyces stipitatus.

## GENOME ANNOUNCEMENT

Penicillium marneffei is the third most prevalent opportunistic infectious microbe of HIV positive patients in northern Thailand and is associated with a high mortality rate (1). P. marneffei was originally identified in 1956 from a bamboo rat in Vietnam (2). The fungus is dimorphic, growing as filaments at 25°C and as yeast at 37°C. Human P. marneffei infection is presumed to result from inhalation of airborne environmental conidia, but this has never been proven. The natural reservoir of P. marneffei that causes human infections remains unidentified. The fungus has only been isolated from human patients and from or associated with 4 species of bamboo rats (3). While analysis of human and bamboo rat isolates have demonstrated that both hosts can share the same strains, the geographically local populations of strains are highly clonal, even though P. marneffei can likely reproduce sexually (4, 5). Unlike other dimorphic fungal pathogens, P. marneffei is most closely related to Talaromyces species (6) with Talaromyces stipitatus being the most closely related homothallic relative. P. marneffei was recently renamed Talaromyces marneffei following a genus-wide reclassification of species (7).

The genome sequences of the type strains of P. marneffei ATCC18224 and T. stipitatus ATCC10500 were determined using the whole-genome shotgun method as described in reference (8). Random shotgun libraries of 2- to 3-kb, 8- to 12-kb, and 50-kb insert sizes were constructed from genomic DNA of each strain, and a DNA template was prepared for high-throughput sequencing using the ABI 3730XL instrument. Sequence reads were assembled using Celera Assembler. Protein-coding genes were annotated using the J. Craig Venter Institute (JCVI) eukaryotic annotation pipeline as described in reference 9.

For P. marneffei ATCC18224, 295,780 paired-end Sanger reads were assembled into 589 contigs. Of these, 295 contigs were incorporated into 20 scaffolds greater than 2 kb in size. The genome size was determined to be 28.5 Mb. Genome coverage was 8.6×. Since penicilliosis fatalities result from liver failure as the fungus releases mycotoxins into the bloodstream, we determined the number of secondary metabolite biosynthetic gene clusters in the genome using the informatics tool SMURF (10). Forty-eight such clusters were found for this strain suggesting that the fungus is capable of producing at least forty-eight secondary metabolites. Aspergillus fumigatus, which produces gliotoxin that can be detected in blood during an invasive infection, contains 35 such biosynthetic clusters (10).

For T. stipitatus ATCC10500, 352,456 paired-end Sanger reads were assembled into 960 contigs. The assembly generated 39 scaffolds greater than 2 kb in size. The genome size was determined to be 35.6 Mb. Genome coverage was 8.1×. The genome encodes 61 secondary metabolite biosynthetic gene clusters, comparable to the numbers found in Aspergillus niger and Aspergillus terreus (10).

### Nucleotide sequence accession numbers.

The annotated genome sequence of P. marneffei ATCC18224 has been deposited at GenBank under the accession numbers DS995899 and DS996350. The whole-genome shotgun (WGS) master record accession number is ABAR00000000. The annotated genome sequence of T. stipitatus ATCC10500 has been deposited under accession number EQ962652 and EQ963471. The WGS master record accession number is ABAS00000000.
